# Tumour Endoprosthetic Reconstruction for Primary Aggressive and Malignant Bone Tumours of the Distal Femur

**DOI:** 10.5704/MOJ.1311.007

**Published:** 2013-11

**Authors:** DA Rubio, MVT Serrano, EHM Wang

**Affiliations:** Department of Orthopedics, Philippine General Hospital, Manila, Philippines; Department of Orthopedics, Philippine General Hospital, Manila, Philippines; Department of Orthopedics, Philippine General Hospital, Manila, Philippines

## Abstract

At the Philippine General Hospital, tumour endoprostheses have become an option for reconstruction after limb saving surgery for primary bone tumors. We performed a retrospective review of patients with primary bone tumors of the distal femur who underwent tumor excision and reconstruction using tumor endoprostheses. Outcome measures included prosthetic survival, functional outcome and complications. Twenty-two patients were evaluated; 14 males and 8 females, with a mean age of 18 years and a mean follow-up of 56 months. The overall 2-year endoprosthetic survival rate was 86%. Mean MSTS was 23/30. There were a total of 6 revisions. Failure modes included 3 infections, 3 aseptic loosening, 4 structural failures, 2 soft tissue failures and 3 tumor progression. Our early results show that tumor endoprosthesis reconstruction is an acceptable option for patients with primary bone tumor of the distal femur. Survival rates, failure modes and functional outcomes are comparable to other reported studies.

**Key Words:**

Supracondylar humerus, K-wire fixation, day care procedure

## Introduction

A variety of reconstructive methods are available after limb saving surgery for malignant and aggressive bone tumors. These include both biologic and non-biologic alternatives such as tumor megaprostheses or endoprostheses. The latter are metal reconstructions which replace not only the entire segment of resected bone but also the adjacent joint. Tumor endoprostheses have been shown to provide both satisfactory functional outcomes and reliable oncologic results 1.

At the University of the Philippines Musculoskeletal Tumor (UP-MuST) Unit of the Philippine General Hospital, the ratio between limb saving surgery and amputation for malignant and aggressive bone tumors is approximately 60% to 40%^2^. Reconstruction after limb salvage has conventionally been restricted to biologic options, such as autografts and allografts. Endoprosthetic reconstructions (EPR) have become more frequent in recent years because of their increasing availability and affordability. We present the survivorship, functional outcome and failure modes of tumor EPR of the distal femur in our institution.

The objective of this study was to determine survivorship, functional outcome and failure modes among patients with bone tumours of the distal femur who underwent tumor excision and reconstruction using tumor endoprosthesis at the UP-MuST Unit of the Philippine General Hospital.

## Materials and Methods

A retrospective review of the files of patients with primary aggressive and malignant bone tumors of the distal femur who had undergone tumor excision and tumor EPR was undertaken. Data was obtained from the UP-MuST unit database at the Department of Orthopedics, Philippine General Hospital, the tumor registry, and the records of the senior author.

Patients with histologically confirmed diagnosis of primary aggressive or malignant bone tumour of the distal femur were included in the study. They must have undergone wide excision of the tumor followed by reconstruction with a tumor endoprosthesis (whether in the same stage or as a second staged procedure), with a follow-up of at least 2 years. Post-op regimen included 2 weeks on knee immobilizer, followed by gradual range of motion, partial to full weight bearing as tolerated on crutches. Post-op rehabilitation program was always supervised by a licensed physical therapist. Patients must have had complete treatment by the UP-MuST. All consecutive patients who met these criteria were included in the study.

Over a period of 11 years (1999-2010), a total of 30 patients underwent tumour EPR for primary aggressive or malignant bone tumors of the extremities. Bones involved were the distal femur (22), proximal tibia (4), proximal humerus (2), and proximal femur (2). We limited this study to patients with primary aggressive and malignant bone tumour of the distal femur in order to evaluate a homogenous population.

Charts of patients who fulfilled the above criteria were retrieved and data recorded into a Data Collection Form (DCF). Patients who were last seen in clinic more than 3 months earlier were contacted for follow-up and physical examination and radiographs repeated.

The study was approved by the Ethics Review Board of our institution.

DCF contained the patient’s age, contact details, diagnosis, laterality, Enneking stage, procedures done, neoadjuvant therapy, extent of soft tissue and bone resection, failure modes and latest revised Musculoskeletal Tumor Society score 3. Data was kept confidential and anonymous.

Functional scores were based on the revised Musculoskeletal Tumor Society rating scale. Six parameters — pain, function, use of supports, walking ability, gait, and emotional acceptance — were evaluated and graded from 0 to 5 points. The scores for each of the parameters were added to obtain the overall functional score, which was expressed as a percentage of the total possible score of 30 points 3. A goniometer was used for documenting range of motion.

Failure modes were based on the classification proposed by Henderson et.al 4. They identified and classified failure modes into five types: 1-soft tissue failures, 2-aseptic loosening, 3-structural failures, 4-infection and 5-tumor progression. Soft tissue failures included instability, tendon rupture or wound dehiscence. Aseptic loosening is described as clinical and radiographic evidence of prosthetic loosening. Structural failures refer to periprosthetic or prosthetic fracture or deficient osseous supporting structure. A classification of infection was made if it was necessary to remove the endoprosthetic device as a result of infection. Tumor progression is a recurrence or progression of tumor with contamination of the endoprosthesis 4.

Survivorship of the tumor prosthesis was evaluated and failure was defined as patients continuing to have severe pain, patients who underwent revision of the prosthesis, or patients who eventually required amputation for a complication 5.

## Results

**Fig. 1:**
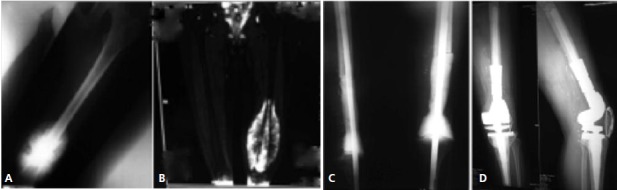
: A. knee radiograph of a patient with osteosarcoma of the distal femur. B. MRI of the distal femur in the same patient C. 1st stage reconstruction – resection of tumor, application of kunstcher nail and cement spacer D. Second stage tumor endoprosthesis reconstruction.

A total of 22 patients were included and evaluated: 14 males and 8 females. Ten patients underwent a single stage procedure (tumor excision and endoprosthetic reconstruction in a single surgery). Twelve (12) patients underwent a 2- stage reconstruction, with the first stage being resection of tumor, application of a long intramedullary Kuntscher nail and a cement spacer around the nail (figure 1). Mean age was 18 years (9 to 65), and mean follow-up was 56 months (24 to 180 months). Diagnoses included 20 patients with classic high grade osteosarcoma and 2 patients with Giant Cell Tumor of bone. All osteosarcoma patients received at least 3 cycles of neoadjuvant chemotherapy prior to tumor EPR followed by another 3 courses of postoperative chemotherapy. For those patients who underwent staged EPR, chemotherapy had already been completed prior to the second reconstructive surgery.

The following companies provided the endoprostheses used: United Orthopedic Corporation (20), MRS How Medica (1), and Biomet Finn Prosthesis (1).

On latest follow-up, nineteen patients were alive with no evidence of disease. Average follow-up for these patients was 55 months. Three patients had died of disease, all having developed pulmonary metastases. One of these three patients developed a metachronous lesion in the proximal tibia; this patient was treated with a hip disarticulation. Another had a local recurrence over the greater trochanter and underwent wide resection and reconstruction with a total femur EPR.

Clinical results were based on the revised Musculoskeletal Tumor Society (MSTS) rating score on the patient’s latest follow-up. The mean MSTS score for the 22 patients was 23/30 (range 17 to 30/30). There was a high limb salvage rate of 95% (21/22), one patient underwent hip disarticulation for a metachronous tibial lesion of the ipsilateral extremity. The mean knee flexion in our series was 94 degrees. We have no findings or reports of knee instability.

The overall 2-year survival rate was 86%. There were a total of 6 revisions (27%), the causes of which were structural failures in 5 and tumour progression in one.

Failure modes were classified according to that of Henderson et al 4.

## Discussion

The distal femur is a common anatomic location for both primary malignant and aggressive bone tumors such as classic high grade osteosarcoma and Giant Cell Tumor of bone^6^. In the past, these tumors were often treated with either amputation or resection arthrodesis, both of which resulted in limitations of movement and function 1. Tumor EPR was developed to allow restoration not only of the segment of bone removed but also the adjacent osteoarticular defects 7.

In the past decade, these tumor EPR have become more popular in the Philippines because of their increasing availability, especially with the recent influx of more affordable Asian brands on the market. This paper is an evaluation of our early results with EPR for limb salvage surgery. We have chosen to limit our population to those patients with tumors of the distal femur alone in order to have a homogenous population.

The overall limb salvage rate for our 22 patients was 95%, one patient requiring a hip disarticulation for a metachronous osteosarcoma lesion in the ipsilateral leg. Seventeen out of the 20 (85%) osteosarcoma patients and both GCT patients were alive with no evidence of disease at latest follow-up. The mean oncologic follow-up was 55 months with a range of 19-180 months.

The mean revised MSTS score was 23/30 (77%). This is similar to the results of large series from Malawer, Bickels and Ahlman. (Table II). Of the 6 parameters of the revised MSTS rating score, function was the lowest scoring parameter. This may be attributed to reduced muscle bulk and function resulting from the radical dissection required for wide tumor margins 8.

Ten patients received their EPR during the same surgery as the tumor excision while 12 patients had their EPR at a later date, usually 6-12 months after end of treatment. The 2 year survival rate for the 1-stage group was 70%, while that of the 2-stage group was 100%. The mean MSTS for the 1-stage group was 24.5/30 (82%) and for the 2 stage group, 23/30 (77%).

Henderson et al recently compiled EPR data (n = 2174) from 5 large tumor centers in Europe and North America and classified failure modes after EPR into 5 types: Type 1-soft tissue failures (12%), Type 2-aseptic loosening (19%), Type 3-structural failures (17%) , Type 4-infection (34%) and Type 5-tumor progression (17%). These results were not very different from that in literature: aseptic loosening (5- 27%), fractures of the stem or adjacent bone (1%-22%), periprosthetic infection (1%-36%), and local recurrence rate (1%-9%) 9-11. However, in the series of Henderson et al, the most common failure mode was infection; while in literature, aseptic loosening was more common 4.

In our series, the most common failure mode was structural failure (18%). This may be attributed to the increased incidence of patellar fractures. Out of our 4 structural failures, 2 patients had patellar fractures, one of which was an intraarticular resection in which the patella was cut on a coronal plane. In both fractures, the patella was resurfaced. In resurfaced patellae, fracture prevalence ranges from 0.2 to 21% 13 Furthermore, most of our patients belong to the pediatric age group, the patella of these patients are small and thin; this adds to the risk of fracture.

The lower incidence of aseptic loosening may be explained by the relatively shorter duration of follow-up compared to several series published in literature 1,7-8,14. Time to failure by aseptic loosening in the distal femur is observed to be 75+/- 62 months 4. Our follow-up ranges from 19-180 months with an average of 55 months. Furthermore, Henderson et al observed that technological advances in the detection of latent infections may have curtailed the onset of loosening that would have been judged previously to be aseptic 4.

On the other hand, our lower infection rate may be partially due to the practice of a delayed second stage EPR in more than half of our patient population. In this 2 stage reconstruction, patients receive, after tumor excision, a temporary reconstruction consisting of an intramedullary nail and a spacer of antibiotic-impregnated bone cement shaped into the size of the resected distal femur. The underlying reason is most often the cost of the endoprothesis, an expense not shouldered by any government or local health insurance. Postoperatively patients complete their remaining chemotherapy courses. There is then a delay of approximately 6-12 months before the actual EPR, by which time the patient has recovered both medically and financially. This delayed second stage removes the chance of doing surgery in between chemotherapeutic sessions with the associated neutropenia and decreased immunogenic response 15.

**Table I:**
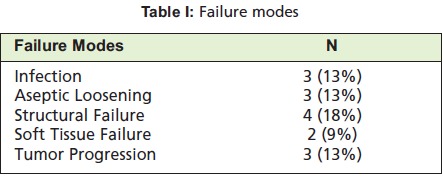
: Failure modes

**Table II:**
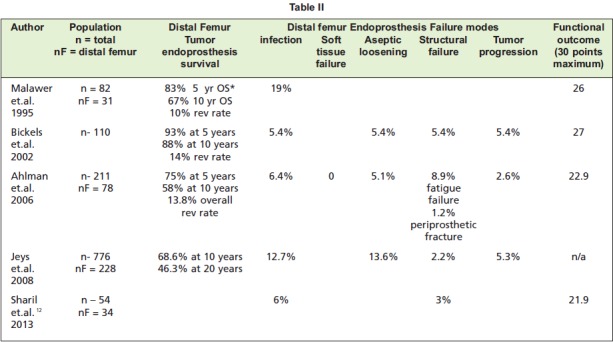


**Fig. 2:**
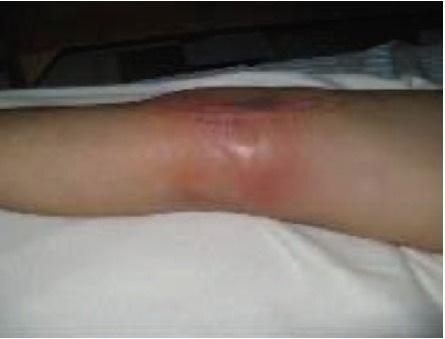
: Infection.

**Fig. 3:**
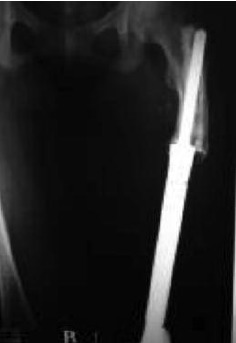
: Aseptic Loosening

**Fig. 4:**
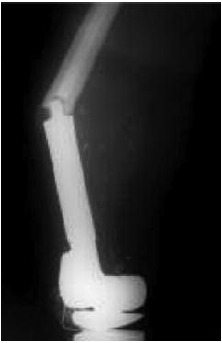
: Structural Failure

## Conclusion

Early results of our distal femoral tumor endoprosthetic reconstruction are encouraging, with survival rates, functional outcome and failure modes comparable to those in literature. EPR provides a good option for limb salvage reconstruction in our patients with aggressive and malignant bone tumors of the distal femur.
